# Hemoglobin and Endotoxin Levels Predict Sarcopenia Occurrence in Patients with Alcoholic Cirrhosis

**DOI:** 10.3390/diagnostics13132218

**Published:** 2023-06-29

**Authors:** Akihiko Shibamoto, Tadashi Namisaki, Junya Suzuki, Takahiro Kubo, Satoshi Iwai, Fumimasa Tomooka, Soichi Takeda, Yuki Fujimoto, Takashi Inoue, Misako Tanaka, Aritoshi Koizumi, Nobuyuki Yorioka, Takuya Matsuda, Shohei Asada, Yuki Tsuji, Yukihisa Fujinaga, Norihisa Nishimura, Shinya Sato, Hiroaki Takaya, Koh Kitagawa, Kosuke Kaji, Hideto Kawaratani, Takemi Akahane, Akira Mitoro, Hitoshi Yoshiji

**Affiliations:** 1 Department of Gastroenterology, Nara Medical University, 840 Shijo-cho, Kashihara 634-8521, Nara, Japan; 2 Department of Evidence-Based Medicine, Nara Medical University, 840 Shijo-cho, Kashihara 634-8521, Nara, Japan

**Keywords:** sarcopenia, alcoholic cirrhosis, nonalcoholic cirrhosis, hemoglobin, endotoxin activity assay

## Abstract

Alcohol is a major risk factor of liver cirrhosis (LC). This study aimed to elucidate a surrogate marker of sarcopenia in patients with LC of different etiology. Out of 775 patients with LC, 451 were assessed for handgrip strength and skeletal muscle mass (by computed tomography). They were then divided into two groups: alcoholic cirrhosis (AC; *n* = 149) and nonalcoholic cirrhosis (NAC; *n* = 302). Endotoxin activity (EA) levels were measured with an EA assay. Group AC showed significantly higher platelet counts (*p* = 0.027) and lower blood urea nitrogen levels and fibrosis-4 index than group NAC (*p* = 0.0020 and *p* = 0.038, respectively). The risk factors of sarcopenia were age ≥ 65 years, female sex, CP-C LC, Hb levels < 12 g/dL, and EA level > 0.4 in all patients with LC; hemoglobin (Hb) levels < 12 g/dL and EA level > 0.4 in group AC; and age ≥ 65 years, CP-C LC, and Hb levels < 12 g/dL in group NAC. The prediction accuracy of Hb for sarcopenia in group AC, group NAC, and all patients was 83.6%, 75.9%, and 78.1% (sensitivity: 92.0%, 69.0%, and 80.2%; specificity: 66.4%, 71.0%, and 64.0%), respectively. Although not significant, the predictive performance was better when using the combination of Hb and EA measurements than when using Hb alone in group AC but was comparable in all patients. Hb levels can predict sarcopenia in patients with LC, but in those with AC, the combination of Hb and EA improves the prediction performance.

## 1. Introduction

Liver cirrhosis (LC) has become one of the major causes of morbidity and mortality in the world [1,2]. Alcohol abuse is responsible for more than 50% of mortality of LC worldwide and remains a significant global problem [3]. In Japan, although cases of viral hepatitis-related LC decreased, those of alcohol-related disease (ALD) and nonalcoholic steatohepatitis (NASH)-related LC notably increased [4]. Nonetheless, the prognosis of patients with LC has improved because of the current management of LC complications, including sarcopenia [5,6].

Sarcopenia refers to progressive and generalized loss of skeletal muscle mass and strength and indicates a poor prognosis in patients with LC [7,8]. In LC, sarcopenia is multifactorial and cannot be fully explained by simple malnutrition [9]. Several mechanisms are involved in the development of sarcopenia in LC, many of which are secondary to deteriorated liver function in treating LC. Hyperammonemia, increased myostatin, and decreased anabolic and growth hormones contribute to imbalance in the synthesis and degradation of muscle proteins [10]. The prevalence of sarcopenia was influenced by age, gender, the etiology and the severity of LC [11]. Recently, low hemoglobin levels have been associated with muscular strength [12]. Anemia has a negative impact on mortality, disability, and physical performance [13]. A causallinkage between skeletal oxygen consumption and blood flow has been previously reported [14]. Low hemoglobin levels have been independently identified as a risk factor for patients with LC, with increased odds of hepatic decompensation and mortality [15]. The pathogenesis of sarcopenia in LC should be further elucidated to produce more effective drug therapies. Body composition was assessed by computed tomography (CT) and bioelectrical impedance analysis (BIA) to diagnose sarcopenia. However, these devices may not be optimal as routine clinical tools because of time, cost, and radiation exposure. A simple, economical, noninvasive tool for the diagnosis of sarcopenia was developed for patients with LC.

## 2. Methods

### 2.1. Patients

**Figure 1:**
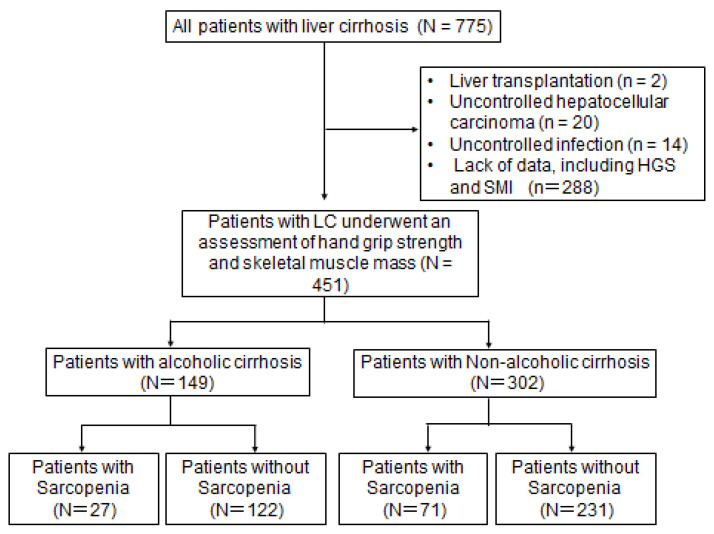
Flowchart of the current study.

This single-center cohort retrospective study included outpatients with LC who visited the Nara medical university hospital between January 1990 and March 2022. Out of 775 patients, 324 were excluded because of the following reasons: liver transplantation (*n* = 2), uncontrolled hepatocellular carcinoma (*n* = 20), uncontrolled infection (*n* = 14), and lack of data including laboratory tests, handgrip strength (HGS) measurement, or CT images (*n* = 288; Figure 1). A total of 451 consecutive patients with LC were ultimately included. LC was diagnosed according to clinical data such as laboratory tests (e.g., albumin, bilirubin, and prothrombin time), medical imaging features, liver histology, and clinical complications (e.g., hepatic encephalopathy and ascites). The etiological classification of LC was as follows: (1) Hepatitis viral infection (caused by hepatitis B virus (HBV); *n* = 43, 9.5% and hepatitis C virus (HCV); *n* = 126, 27.9%); (2) ALD (*n* = 149, 33.1%); (3) nonalcoholic steatohepatitis ((NASH); *n* = 76, 16.9%); and (4) others including autoimmune hepatitis (AIH), primary biliary cirrhosis, and idiopathic causes (*n* = 57, 12.6%). Hepatitis viral infection was diagnosed based on the generally accepted serological criteria, including positivity for HBs antigen for the diagnosis of HBV infection and positivity for both HCV antibodies and HCV RNA. ALD is currently diagnosed based on the history of alcohol consumption (alcohol intake ≥ 60 g/day), physical examination, and laboratory tests. NASH is diagnosed by conducting a liver biopsy. In this study, alcohol intake was the leading cause of LC. Therefore, patients were divided into two groups: patients with alcoholic cirrhosis ((AC); *n* = 149) and those without AC ((NAC); *n* = 302). All patients with LC received dietary advice and a verbal explanation from the nutritionist. Dietary protein intake showed no remarkable differences in individual patients. No patients underwent parenteral or oral iron supplementation. In addition, the attending physicians encouraged patients with AC to completely abstain from alcohol. The study protocol conformed to the ethical guidelines of the Declaration of Helsinki. The Medical Ethics Committee of Nara Medical University approved our study protocol (Nara-medi, 1352-12-5). All patients provided written informed consent to blood sampling before study enrollment.

A: A total of 775 outpatients with liver cirrhosis (LC) were enrolled. To evaluate the general trends in the etiologies of LC, we stratified the patients into six groups (2007, 2008–2010, 2011–2013, 2014–2016, 2017–2019, and 2020–2022). B: We retrospectively reviewed the medical records of 451 patients with LC who were assessed on handgrip strength (HGS) and skeletal muscle mass (via computed tomography). Out of 775 patients, 39 were excluded due to the following reasons: liver transplantation (*n* = 2), uncontrolled hepatocellular carcinoma (*n* = 20), uncontrolled infection (*n* = 14), and lack of data including laboratory tests, HGS measurement, or computed tomography images (*n* = 288).

### 2.2. Diagnosis of Sarcopenia

The Japanese Society of Hepatology (JSH) established the original criteria for liver disease-related sarcopenia according to the Asian criteria for sarcopenia in 2016. In this study, sarcopenia was diagnosed using the Assessment Criteria for Sarcopenia in Liver Disease (2nd edition updated in 2020) according to the Sarcopenia Assessment Criteria of the JSH [16].

HGS was assessed using a handheld dynamometer [17]. The amount of skeletal muscle mass was retrospectively defined using the skeletal muscle mass index (SMI), which was calculated by measuring the skeletal muscle at the level of the third lumbar (L3) vertebra on CT images (CT-SMI). Patients were diagnosed with sarcopenia if they had low muscle strength (male < 28 kg, female < 18 kg) plus low muscle mass by CT (male < 42 cm^2^/m^2^, female < 38 cm^2^/m^2^) or low BIA (male: <7.0 kg/m^2^, female: 5.7 kg/m^2^) according to the JSH criteria [18].

### 2.3. Myostatin Endotoxin Activity Assay (EAA) Measurements

A serum assay for endotoxin activity (EA) was evaluated in all of the 451 patients with LC. EAA was used to measure the EA levels according to the assay manufacturers’ protocols (Toxicolor LS-50-M Set; Seikagaku Corp., Tokyo, Japan) [19,20]. Serum myostatin concentrations were measured twice using commercially available kits (DGDF80; R&D Systems, Minneapolis, MN, USA). Intra- and inter-assay coefficients of variation were less than 10% [21].

### 2.4. Statistical Analyses

All statistical data were analyzed using R software, version 4.0.2 (The R Foundation for Statistical Computing, Vienna, Austria). Normally and non-normally distributed continuous variables are expressed as mean ± standard deviation and median (interquartile range of 25–75%), respectively. Categorical variables are presented in a contingency table. The baseline characteristics between groups were compared using the unpaired *t*-test or the Mann–Whitney *U* test. Parametric tests for continuous data with a normal distribution, and nonparametric tests for those without a normal distribution were used. To concurrently evaluate the effects of multiple risk factors in survival time analysis, the logistic regression model was used. Parameters in with a *p* value of <0.05 were included in the linear regression model. Additionally, categorical data were analyzed using Fisher’s exact test. A two-sided *p* value of <0.05 indicated statistical significance. Although an explanatory variable remains a continuous variable, it is possible to calculate the relationship between the explanatory and objective variables. However, in many cases, the relationship between explanatory and objective variables is not linear. This relationship is frequently used after classifying the explanatory variable into some categorical groups consisting of two or more categories using appropriate threshold values that have been optimized in some way. The groups generated based on the explanatory variable will have medical meaning, e.g., a high-risk group or a low-risk group for a given disease. Furthermore, the grouping thresholds are expected to function as useful indicators for both medical practitioners and patients, considering that these markers can provide an estimate of the probability of a disease before any test is ordered.

## 3. Results

### 3.1. Prognostic Differences among Patients with Different Etiologies and Hepatic Function Reserve

**Figure 2:**
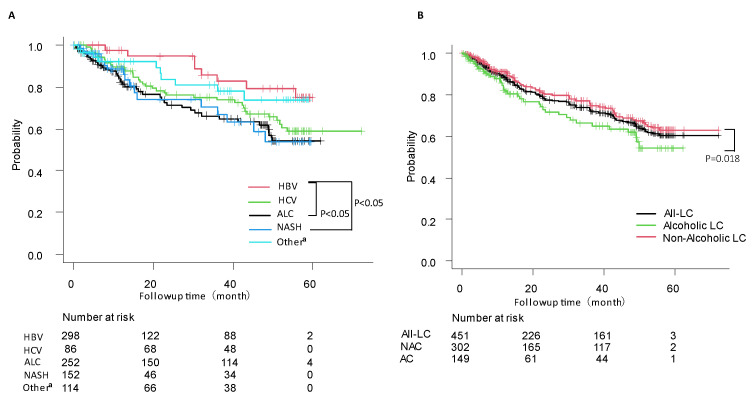
Kaplan–Meier curve analysis. Survival curves stratified by (**A**) etiology and (**B**) patients with AC, patients with nonalcoholic cirrhosis, and all patients. AC, alcoholic cirrhosis; NAC nonalcoholic cirrhosis. ^a^ Includes autoimmune hepatitis, primary biliary cholangitis.

The overall survival rates were significantly better in patients with HBV than in those with AC and NASH-related cirrhosis (*p* = 0.013 and *p* = 0.031) (Figure 2A). Furthermore, the overall survival rates were higher in group AC than in group NAC (*p* = 0.018) (Figure 2B).

### 3.2. Comparison of Clinical Characteristics between Groups AC and NAC

Table 1 summarizes the baseline clinical characteristics and laboratory data of all patients. Out of 451 patients with LC, 288 (63.9%) were male, and the mean age and body mass index (BMI) of the total study cohort were 69.1 ± 10.8 years and 24.3 ± 4.5 kg/m^2^, respectively. Males had higher mean HGS (29.8 ± 8.7 vs. 16.1 ± 6.3) and mean CT-SMI (46.0 ± 8.9 vs. 39.2 ± 8.8) than females. The mean Child–Pugh (CP) score was 6.1 ± 1.6, with 334 (74.1%), 97 (21.5%), and 20 (4.4%) patients classified into CP classes A, B, and C, respectively. Regarding the albumin–bilirubin score (ALBI), 194 (43.0%), 89 (19.7%), 136 (30.2%), and 32 (7.1%) patients were stratified into 1, 2a, 2b, and 3, respectively. Moreover, the mean fibrosis-4 (FIB-4) index was 5.8 ± 4.5. Group NAC consisted of 43 patients with HBV, 126 with HCV, 76 with NASH, and 57 with other disorders, including primary biliary cholangitis, autoimmune hepatitis, and idiopathic causes in 23, 29, and 5 patients, respectively. Group AC was significantly younger and had significantly fewer females than group NAC (*p* < 0.001 and *p* < 0.001, respectively). In addition, group NAC had significantly lower platelet counts (*p* = 0.027) but had higher levels of blood urea nitrogen (BUN) and FIB-4 than group AC (*p* = 0.0020 and *p* = 0.038, respectively).

Table 2 shows the clinical differences of all patients with and without sarcopenia. Patients with sarcopenia were significantly older and consisted of significantly fewer males than those without sarcopenia (both: *p* < 0.001). The proportion of all patients who had CP-A LC or ALBI grade 1/2a was significantly higher in patients without sarcopenia than in those with sarcopenia (both: *p* < 0.001). Significantly lower indices were observed in patients with sarcopenia in terms of BMI, HGS in both males and females, SMI in both males and females, Hb levels in both males and females, and BTR as compared with those without sarcopenia (*p* < 0.001, *p* < 0.001, *p* < 0.001, *p* < 0.001, *p* < 0.001, *p* < 0.001, *p* < 0.001, and *p* = 0.022, respectively). Those with sarcopenia also had significantly lower serum levels of albumin, cholinesterase, sodium, and zinc than those without (*p* < 0.001, *p* < 0.001, *p* = 0.0027, and *p* = 0.0018, respectively). CPS, ALBI score, ascites incidence, and the serum levels of BUN, P-III-P, myostatin, and EA were significantly higher in those with sarcopenia than in those without (*p* < 0.001, *p* < 0.001, *p* = 0.011, *p* = 0.028, *p* < 0.001, *p* = 0.018, and *p* = 0.024, respectively).

### 3.3. Comparison of Clinical Characteristics between Patients with LC with and without Sarcopenia

The overall prevalence of sarcopenia was 21.7% (98/451) in all patients with LC, 18.1% (27/149) in group AC, and 23.5% (71/302) in group NAC. Table 3 shows the clinical differences of patients with and without sarcopenia in group AC. The proportion of group AC who had ALBI grade 1/2a was significantly higher in patients without sarcopenia than in those with sarcopenia (*p* = 0.040). Significantly lower indices were observed in those with sarcopenia in terms of BMI, HGS in males, CT-SMI in both males and females, and Hb levels in both males and females as compared with those without sarcopenia (*p* = 0.0063, *p* < 0.001, *p* < 0.001, *p* = 0.016, *p* < 0.001, and *p* = 0.048, respectively). Furthermore, the serum levels of albumin, cholinesterase, sodium, and zinc were significantly lower in those with sarcopenia than in those without sarcopenia (*p* < 0.001, *p* < 0.001, *p* = 0.0025, and *p* = 0.030, respectively). Those with sarcopenia also had significantly higher CPS, ALBI score, BUN level, procollagen N-terminal peptide (P-III-P) level, myostatin level, and EA level than those without sarcopenia (*p* = 0.0041, *p* = 0.0038, *p* = 0.0054, *p* = 0.0066, *p* = 0.035, and *p* < 0.001, respectively).

Table 4 shows the clinical differences of patients with and without sarcopenia in group NAC. Patients with sarcopenia were significantly older and consisted of fewer males than those without sarcopenia (*p* < 0.001 and *p* = 0.0096, respectively). The proportion of group NAC who had CP-A LC or modified ALBI grade 1/2a was significantly higher in patients without sarcopenia than in those with sarcopenia (*p* = 0.0022 and *p* = 0.0042). Significantly lower indices were observed in patients with sarcopenia in terms of BMI, HGS in both males and females, SMI in both males and females, Hb levels in both males and females, and branched amino acids-to-tyrosine ratio (BTR) as compared with those without sarcopenia (*p* < 0.001, *p* < 0.001, *p* < 0.001, *p* < 0.001, *p* < 0.001, *p* = 0.0022, *p* < 0.001, and *p* = 0.030, respectively). The serum levels of albumin, cholinesterase, and zinc were significantly lower in patients with sarcopenia than in those without (*p* < 0.001, *p* < 0.001, and *p* = 0.024, respectively). CPS, ALBI score, gastroesophageal varices incidence, and serum P-III-P levels were significantly higher in those with sarcopenia than in those without (*p* = 0.0011, *p* < 0.001, *p* = 0.002, and *p* = 0.018, respectively).

### 3.4. Risk Factors for Sarcopenia

**Figure 3:**
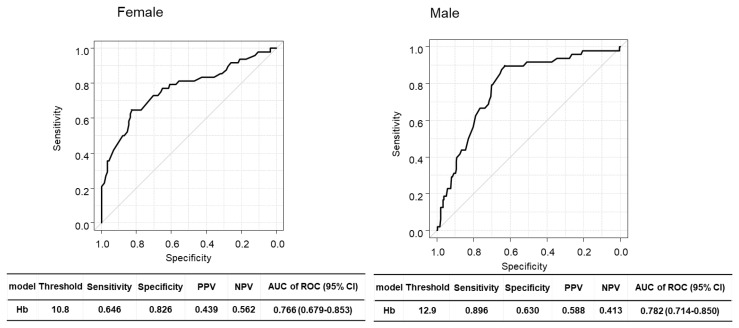
Receiver-operating characteristic curve analysis of hemoglobin levels in relation to sarcopenia in female and male patients with liver cirrhosis.

We investigated the risk factors for sarcopenia in patients with LC. The optimal cutoff values of Hb levels were 10.8 g/dL in females and 12.9 g/dL in males for diagnosing sarcopenia (Figure 3). In all patients with LC, the multivariate analysis identified five predictors of sarcopenia: age ≥ 65 years (hazard ratio (HR) 2.39, 95% confidence interval (CI) 1.14–5.01, *p* = 0.0021), CP-C LC (HR 3.80, 95% CI 1.05–13.80, *p* = 0.042), Hb levels <10.8 g/dL females, Hb levels < 12.9 g/dL males (HR 4.47, 95% CI 2.32–8.60, *p* < 0.001) (Table 5). Hb levels < 10.8 g/dL in females and < 12.9 g/dL in males (HR 5.97, 95% CI 1.88–19.00, *p* = 0.0025), and EA level > 0.4 (HR 5.02, 95% CI 1.60–15.80, *p* = 0.0057) were identified as the independent risk factors of sarcopenia in group AC (Table 6). Age ≥ 65 years (HR 3.09, 95% CI 1.21–7.87, *p* = 0.018), CP-C LC (HR 4.10, 95% CI 1.09–15.50, *p* = 0.037), gastroesophageal varices incidence (HR 2.11, 95% CI 1.06–4.20, *p* = 0.033), and Hb levels < 10.8 g/dL females, Hb levels < 12.9 g/dL males (HR 5.34, 95% CI 2.52–11.30, *p* < 0.001) were identified as the independent risk factors for sarcopenia in group NAC (Table 7).

### 3.5. Prediction Accuracy of Hb Levels for Sarcopenia

**Figure 4:**
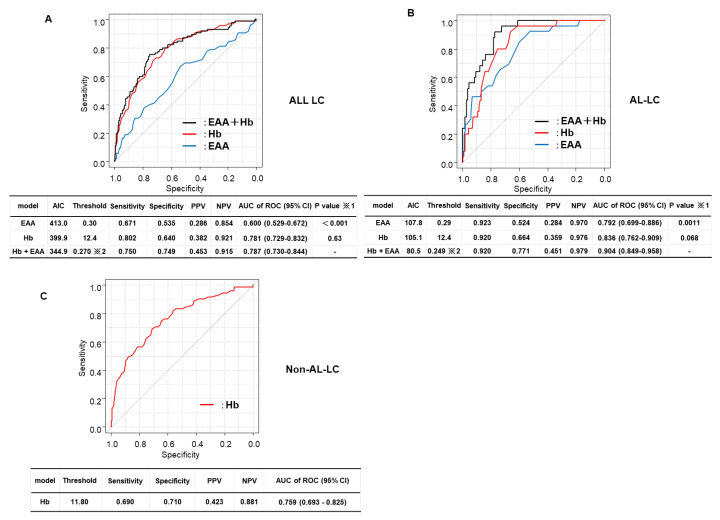
Receiver-operating characteristic curve analysis of hemoglobin and endotoxin activity levels in relation to sarcopenia in patients with liver cirrhosis. (**A**): ROC curves of Hb and EA levels alone and combined for identifying sarcopenia in all patients with LC. The diagnostic accuracy of Hb and EA levels individually and combined for identifying sarcopenia was compared in all patients with LC. (**B**): ROC of Hb and EA levels alone and combined for identifying sarcopenia in patients with AC. (**C**): The diagnostic accuracy of Hb and EA levels individually and combined for identifying sarcopenia was compared in patients with AC. ROC curves of Hb and EA levels alone and combined for identifying sarcopenia in patients with NAC. ROC, receiver-operating characteristic; EA, endotoxin activity; EAA, endotoxin activity assay; AC, alcoholic cirrhosis; NAC, nonalcoholic cirrhosis; Hb, hemoglobin; AIC; Akaike’s information criterion; PPV, positive predictive value; NPV, negative predictive value; AUC, area under the curve; CI, confidence interval. ※1. Comparison of AUC for Hb alone, EAA alone, and their combination was performed using bootstrapping for paired ROC curves. ※2. An index based on the propensity score.

The prediction accuracy of the combination of Hb levels and EA levels for all patients was 78.1% (80.2% sensitivity, 64.0% specificity) and 60.0% (67.1% sensitivity, 53.5% specificity) (Figure 4A). The prediction accuracy of the composite index based on Hb and EA measurements for all patients was 78.7% (75.0% sensitivity, 74.9% specificity), For group AC, the prediction accuracy of Hb levels and EA levels was 83.6% (92.0% sensitivity, 66.4% specificity) and 79.2% (92.3% sensitivity, 52.4% specificity), respectively (Figure 4A), but that of the Hb levels and EA level combined was 90.4% (92.0% sensitivity, 77.1% specificity). The combination of these markers had a significantly better specificity than EA levels alone, but not when compared with Hb levels alone (*p* = 0.0011 and 0.068, respectively). Furthermore, the prediction accuracy of Hb levels for group NAC was 75.9% (69.0% sensitivity, 71.0% specificity) (Figure 4B), indicating that the combined index had a comparable performance with Hb levels alone.

### 3.6. Prediction Accuracy of Hb Levels for HGS and SMI

**Figure 5:**
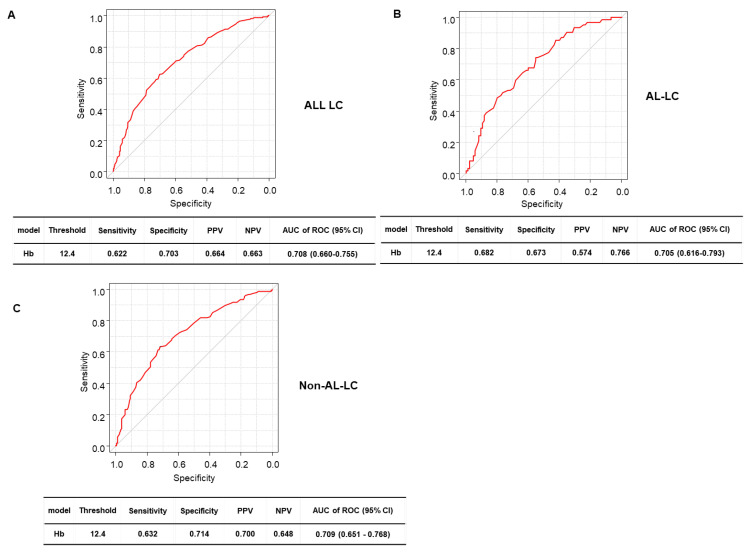
ROC analysis of Hb in relation to handgrip strength in (**A**) all patients with LC, (**B**) patients with AC, and (**C**) those with NAC. ROC, receiver-operating characteristic; AC, alcoholic cirrhosis; NAC nonalcoholic cirrhosis; LC, liver cirrhosis; Hb, hemoglobin; PPV, positive predictive value; NPV, negative predictive value; AUC, area under the curve; CI, confidence interval.

**Figure 6:**
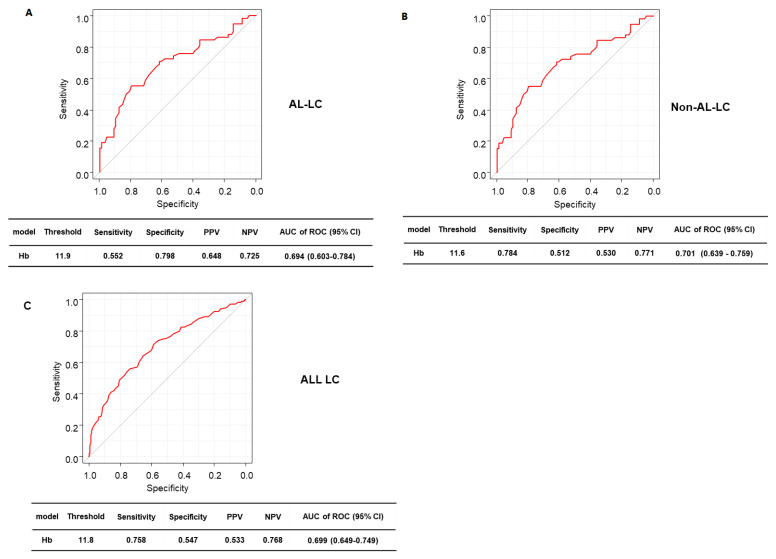
ROC analysis of hemoglobin in relation to skeletal mass index in (**A**) all patients with liver cirrhosis, (**B**) patients with alcoholic cirrhosis, (**C**) those with nonalcoholic cirrhosis. ROC, receiver-operating characteristic; Hb, hemoglobin; PPV, positive predictive value; NPV, negative predictive value; AUC, area under the curve; CI, confidence interval.

The prediction accuracy of Hb levels for HGS in all patients, AC group, and NAC group was 70.8% (62.2% sensitivity, 70.3% specificity), 70.5% (68.2% sensitivity, 67.3% specificity), and 70.9% (63.2% sensitivity, 71.4% specificity) (Figure 5A–C). The overall survival rates for SMI were 69.9% (75.8% sensitivity, 54.7% specificity), 69.4% (55.2% sensitivity, 79.8% specificity), and 70.1% (78.4% sensitivity, 51.2% specificity) (Figure 6A–C), respectively. In addition, the area under the curve of Hb levels was greater for sarcopenia than for HGS and SMI in all patients. 

## 4. Discussion

The prognostic assessment of patients with LC remains challenging. No significant differences in Hb levels were found between the time of sarcopenia diagnosis and 2 years prior. Sarcopenia is associated with significantly higher mortality risk in patients with LC, reaching a prevalence rate of >30% [22], but it is most prevalent in patients with AC [23]. The level of Hb is identified as an independent risk factor associated with sarcopenia in all patients and the two groups. This study is the first to find that Hb level is a noninvasive and reliable tool that can predict the presence of sarcopenia in patients with LC. In group AC, a composite index based on Hb and EA level measurements had significantly better prediction performance than the Hb levels alone. However, their differences were not statistically significant.

Anemia refers to the low number of red blood cells, leading to an insufficient amount of oxygen transported to the skeletal muscle [24]. Impaired oxygen delivery to the muscle contributes to reduced skeletal muscle mass [25]. In addition, Hb levels are associated with skeletal muscle mass in older adults [13,26]. In particular, Hb levels < 14.2 g/dL contribute to sarcopenia development in the population aged ≥ 60 years [27]. Conversely, Hb levels do not affect the skeletal muscle mass in patients with malignancy [26]. This discrepancy among previous studies remains unexplained. However, one possible explanation is differences in the underlying liver disease. In the present study, Hb levels were more likely to predict sarcopenia than HGS or SMI in patients with LC, probably because anemia adversely affected the muscle mass and physical function. Recently, increases in reactive oxygen species and local oxidative stress caused by iron excess were reported to play a pivotal role in muscle atrophy. Furthermore, serum ferritin is negatively associated with skeletal muscle mass [28]. However, our study revealed that iron accumulation does not contribute to sarcopenia pathogenesis. These finding suggest that Hb levels < 12 g/dL can predict the presence of sarcopenia in patients with LC.

The hepatic sinusoid constitutes the microcirculatory bed in the liver parenchyma [29]. The pathogenesis of ALD is characterized by hepatic microcirculatory disturbances [30]. The decrease in microcirculatory hepatic blood flow disrupts the substances between blood and hepatocytes, thereby exacerbating the hepatic injury [31]. Additionally, skeletal muscle capillary rarefaction restricts the transcapillary transport of oxygen to the muscle, possibly contributing to sarcopenia [32]. Therefore, impairment of the skeletal muscle microcirculation causes hypoxia and leads to sarcopenia in patients with cirrhosis, especially those with AC.

This study also found that the EA level was an independent risk factor associated with sarcopenia development in patients with AC. The accuracy of a composite index based on both Hb and EA levels in predicting sarcopenia was more than 0.9 in group AC. The EA levels that reflect intestinal permeability are already increased in patients with AC. However, the effect of acute or chronic ethanol consumption and ethanol withdrawal on the EA levels in patients with AC still remains undetermined. Nonetheless, our findings reinforce the fact that the EA levels improve the diagnostic accuracy of Hb levels for detecting sarcopenia in patients with AC regardless of the frequency and amount of alcohol intake.

In contrast, myostatin levels were not associated with sarcopenia development. An increase in myostatin levels is induced by the multiple factors for LC diagnosis. Myostatin level increase is attributed to decreased levels of serum testosterone and insulin-like growth factor-1 (IGF-1) [33]. Thus, reduced IGF-1 levels represent increased myostatin levels as well as reduced muscle protein synthesis activated by mTOR signaling in patients with LC [33]. Further studies are warranted to evaluate the different risk factors of sarcopenia, categorized into causes of liver disease.

This study acknowledged several limitations. First, it is a prospective, single-center study with a small number of patients, particularly females in group AC. Second, the effects of anabolic hormones, including testosterone, growth hormone, and IGF-1, on sarcopenia occurrence were not examined. Third, all patients with AC were advised to completely stop drinking alcohol; however, there is no confirmation of whether they actually followed this advice.

Taken together, the identification of sarcopenia remains a challenge to be addressed because no simple and rapid diagnostic tool exists in clinical practice. In patients with LC, sarcopenia occurrence can be predicted using the Hb level, which is a simple, cost effective, noninvasive diagnostic tool. In patients with AC, a composite index based on both Hb and EA level measurements improves the prediction performance compared with Hb levels alone. A larger sample size is required to clarify the causal relationship between Hb levels and sarcopenia in patients with LC.

## Figures and Tables

**Table 1:** Clinical characteristics of patients with alcoholic liver cirrhosis and nonalcoholic liver cirrhosis.

Variables	All Patients (*n* = 451)	Alcoholic Cirrhosis (Group AC; *n* = 149)	Nonalcoholic Cirrhosis (Group NAC; *n* = 302)	*p*-Value ^a^
Age, Years	69.1 ± 10.8	66.2 ± 12.0	70.6 ± 9.8	<0.001
Gender, male/female	288/163	135/14	153/149	<0.001
BMI, kg/m^2^	24.3 ± 4.5	24.1 ± 4.49	24.3 ± 4.4	0.53
Child–Pugh grade A/B/C	334/97/20	111/31/7	223/66/13	0.95
Child–Pugh score	6.1 ± 1.6	6.1 ± 1.6	6.1 ± 1.5	0.69
m-ALBI grade 1/2a/2b/3	194/89/136/32	61/34/44/10	133/55/92/22	0.72
ALBI score	−2.38 ± 0.62	−2.38 ± 0.61	−2.38 ± 0.63	0.95
Etiology HCV/HBV/ALC/NAFLD/others ^b^	126/43/149/76/57	149	126/43/76/57	
HCC	105 (23.3)	35 (23.5)	70 (23.2)	0.94
Ascites	79 (17.5)	26 (17.4)	53 (17.5)	0.98
Gastroesophageal varix	224 (49.7)	84 (56.4)	140 (46.4)	0.054
HGS, kg				
Male	29.8 ± 8.7	30.5 ± 9.0	29.2 ± 8.5	0.40
Female	16.1 ± 6.3	17.4 ± 6.7	16.0 ± 6.3	0.21
CT-SMI, cm^2^/m^2^				
Male	46.0 ± 8.9	46.2 ± 9.6	45.9 ± 8.3	0.15
Female	39.2 ± 8.8	36.0 ± 6.8	39.5 ± 8.9	0.83
Sarcopenia	98 (21.7)	27 (18.1)	71 (23.5)	0.23
Hemoglobin, g/dL	12.6 ± 2.1	12.9 ± 2.1	12.5 ± 2.0	0.057
Male	13.1 ± 2.0	13.1 ± 2.0	13.1 ± 2.0	0.94
Female	11.8 ± 1.9	11.1 ± 1.9	11.9 ± 1.9	0.16
MCV, fL	96.0 ± 8.6	96.9 ± 9.1	95.5 ± 8.3	0.098
MCH, pg	31.7 ± 3.4	32.0 ± 3.6	31.5 ± 3.2	0.12
MCHC, %	33.0 ± 1.0	33.0 ± 1.1	32.9 ± 0.9	0.62
Platelet, ×10^4^/μL	11.9 ± 5.6	12.7 ± 5.8	11.5 ± 5.4	0.027
Albumin, g/dL	3.8 ± 0.6	3.9 ± 0.6	3.8 ± 0.7	0.52
Prothrombin time, %	76.5 ± 18.52	74.9 ± 20.2	77.3 ± 17.6	0.20
Total bilirubin, mg/dL	1.2 (0.8–1.7)	1.2 (0.9–1.8)	1.1 (0.8–1.6)	0.14
Cholinesterase, U/L	203.3 ± 91.0	199.2 ± 84.7	205.3 ± 93.9	0.51
BTR	4.8 ± 1.8	4.7 ± 1.8	4.8 ± 1.9	0.87
Ammonia, μg/dL	47.3 ± 28.8	49.8 ± 31.3	46.1 ± 27.5	0.21
BUN, mg/dL	16.4 ± 7.7	14.8 ± 6.5	17.2 ± 8.2	0.0020
Creatinine, mg/dL	0.79 (0.66–0.98)	0.82 (0.66–0.99)	0.78 (0.66–0.97)	0.27
Sodium, mEq/L	139.0 ± 3.5	138.8 ± 3.3	139.1 ± 3.6	0.45
Zinc, μg/dL	67.3 ± 17.0	68.5 ± 16.9	66.8 ± 17.1	0.32
EAA	0.31 ± 0.13	0.30 ± 0.11	0.31 ± 0.14	0.86
P-III-P, U/m	1.0 ± 0.6	1.0 ± 0.5	1.0 ± 0.6	0.98
7S domain of type IV collagen, ng/mL	7.7 (5.7–10.4)	8.2 (6.4–11.5)	7.3 (5.6–10.3)	0.38
FIB4 index	5.8 ± 4.5	5.2 ± 3.4	6.1 ± 4.9	0.038
Ferritin, ng/mL	87.9 (32.0–195.3)	105.4 (34.1–241.0)	80.5 (31.8–170.5)	0.12
CRP, mg/dL	0.10 (0.030–0.50)	0.12 (0.070–0.60)	0.10 (0.020–0.43)	0.80
Myostatin, ng/mL	3.1 ± 1.3	3.0 ± 1.5	3.3 ± 1.1	0.17

Categorical data are presented as number and continuous data are presented as mean (standard deviation) or median value (interquartile range). ^a^ Comparisons of clinical characteristics between alcoholic and nonalcoholic patients were carried out using the χ^2^-test or Student’s *t*-test. ^b^ Includes autoimmune hepatitis, primary biliary cholangitis. BMI, body mass index; HCV, hepatitis C virus; HBV, hepatitis B virus; ALC, alcoholic liver cirrhosis; NASH, nonalcoholic steatohepatitis; HCC, hepatocellular carcinoma; HGS, handgrip strength; SMI, skeletal muscle index; BTR, branched-chain amino acid to tyrosine ratio; BUN, blood urea nitrogen; EAA, endotoxin activity assay; P-III-P, type III procollagen-N-peptide; FIB-4, fibrosis 4 index; MCV, mean corpuscular volume; MCH, mean corpuscular hemoglobin; MCHC, mean corpuscular hemoglobin concentration; PT, prothrombin time; ChE, cholinesterase; NH_3_, ammonia; CRP, C-reactive protein; m-ALBI, modified albumin–bilirubin; CT, computed tomography.

**Table 2:** Clinical characteristics of all patients with sarcopenia and without sarcopenia.

Variables	All Patients (*n* = 451)	Sarcopenia (*n* = 98)	Nonsarcopenic (*n* = 353)	*p*-Value ^a^
Age, Years	69.1 ± 10.8	72.5 ± 10.7	68.2 ± 10.6	<0.001
Gender, male/female	288/163	48/50	240/113	<0.001
BMI, kg/m^2^	24.3 ± 4.5	22.1 ± 3.5	24.9 ± 4.5	<0.001
Child–Pugh grade A/B/C	334/97/20	60/27/11	274/70/9	<0.001
Child–Pugh score	6.1 ± 1.6	6.7 ± 1.9	5.9 ± 1.4	<0.001
m-ALBI grade 1/2a/2b/3	194/89/136/32	26/19/40/13	168/70/96/19	<0.001
ALBI score	−2.38 ± 0.62	−2.12 ± 0.63	−2.45 ± 0.60	<0.001
Etiology HCV/HBV/ALC/NASH/others ^b^	126/43/149/76/57	39/3/27/11/18	87/40/122/65/39	<0.001
HCC	105 (23.3)	28 (28.6)	77 (21.8)	0.18
Ascites	79 (17.5)	26 (26.5)	53 (15.1)	0.011
Gastroesophageal varices	224 (49.7)	56 (57.1)	168 (47.6)	0.17
HGS, kg				
Female	16.1 ± 6.3	12.6 ± 3.9	17.6 ± 6.6	<0.001
Male	29.8 ± 8.7	22.0 ± 4.6	31.4 ± 8.5	<0.001
CT-SMI, cm^2^/m^2^				
Female	39.2 ± 8.8	33.4 ± 3.9	41.8 ± 9.1	<0.001
Male	46.0 ± 8.9	41.2 ± 6.6	47.8 ± 9.0	<0.001
Hemoglobin, g/dL				
Male	13.1 ± 2.0	11.7 ± 1.8	13.3 ± 2.0	<0.001
Female	11.8 ± 1.9	11.0 ± 2.1	12.2 ± 1.6	<0.001
MCV, fL	96.0 ± 8.6	96.7 ± 9.1	95.8 ± 8.5	0.35
MCH, pg	31.7 ± 3.4	31.8 ± 3.6	31.7 ± 3.4	0.83
MCHC, %	33.0 ± 1.0	32.8 ± 1.0	33.0 ± 0.9	0.19
Platelet, ×10^4^/μL	11.9 ± 5.6	12.0 ± 5.8	12.0 ± 5.5	0.82
Albumin, g/dL	3.8 ± 0.6	3.5 ± 0.7	3.9 ± 0.6	<0.001
PT, %	76.5 ± 18.52	73.6 ± 19.1	77.4 ± 18.3	0.074
Total bilirubin, mg/dL	1.2 (0.8–1.7)	1.2 (0.8–1.7)	1.1 (0.8–1.6)	0.43
ChE, U/L	203.3 ± 91.0	152.6 ± 70.9	217.3 ± 91.1	<0.001
BTR	4.9 ± 2.1	4.4 ± 1.6	4.9 ± 1.9	0.022
NH_3_, μg/dL	47.3 ± 28.8	47.2 ± 25.0	47.3 ± 29.9	0.97
BUN, mg/dL	16.4 ± 7.7	17.9 ± 9.1	16.0 ± 7.3	0.028
Creatinine, mg/dL	0.79 (0.66–0.98)	0.79 (0.65–1.03)	0.79 (0.66–0.97)	0.59
Sodium, mEq/L	139.0 ± 3.5	138.1 ± 4.5	139.3 ± 3.2	0.0027
Zinc, μg/dL	67.3 ± 17.0	62.4 ± 20.1	66.7 ± 15.9	0.0018
EAA	0.31 ± 0.13	0.33 ± 0.14	0.29 ± 0.13	0.024
P-III-P, U/m	1.0 ± 0.6	1.2 ± 0.6	1.0 ± 0.5	<0.001
Type IV collagen 7S, ng/mL	7.7 (5.7–10.4)	8.4 (6.7–11.5)	7.5 (5.5–10.3)	0.56
FIB4 index	5.8 ± 4.5	6.2 ± 3.9	5.7 ± 4.7	0.32
Ferritin, ng/mL	87.9 (32.0–195.3)	74.3 (30.3–238.1)	91.0 (32.5–183.3)	0.54
CRP, mg/dL	0.10 (0.030–0.50)	0.27 (0.060–0.72)	0.10 (0.030–0.42)	0.29
Myostatin, ng/mL	3.1 ± 1.3	3.5 ± 1.4	2.8 ± 1.2	0.018

Categorical data are presented as number, and continuous data are presented as mean (standard deviation) or median value (interquartile range). ^a^ Comparisons of clinical characteristics between patients with sarcopenia and without sarcopenia were carried out using the χ^2^-test or Student’s *t*-test. ^b^ Includes autoimmune hepatitis, primary biliary cholangitis. BMI, body mass index; HCV, hepatitis C virus; HBV, hepatitis B virus; ALC, alcoholic liver cirrhosis; NASH, nonalcoholic steatohepatitis; HCC, hepatocellular carcinoma; HGS, handgrip strength; SMI, skeletal muscle index; BTR, branched-chain amino acid to tyrosine ratio; BUN, blood urea nitrogen; EAA, endotoxin activity assay; P-III-P, type III procollagen-N-peptide; FIB-4, fibrosis 4 index; MCV, mean corpuscular volume; MCH, mean corpuscular hemoglobin; MCHC, mean corpuscular hemoglobin concentration; PT, prothrombin time; ChE, cholinesterase; NH_3_, ammonia; CRP, C-reactive protein; m-ALBI, modified albumin–bilirubin; CT, computed tomography.

**Table 3:** Clinical characteristics of alcoholic patients with sarcopenia and without sarcopenia.

Variables	Alcoholic cirrhosis (Group A; *n* = 149)	Sarcopenia (Group S; *n* = 27)	Nonsarcopenic (Group N; *n* = 122)	*p*-Value ^a^
Age, years	66.2 ± 12.0	67.5 ± 15.2	65.9 ± 11.3	0.53
Gender, male/female	135/14	22–5	113/9	0.14
BMI, kg/m^2^	24.1 ± 4.49	21.9 ± 3.8	24.5 ± 4.5	0.0063
Child–Pugh grade A/B/C	111/31/7	16/8/3	95/23/4	0.067
Child–Pugh score	6.1 ± 1.6	6.9 ± 2.2	6.0 ± 1.4	0.0041
m-ALBI grade 1/2a/2b/3	61/34/44/10	6/5/13/3	55/29/31/7	0.040
ALBI score	−2.38 ± 0.61	−2.08 ± 0.63	−2.45 ± 0.58	0.0038
HCC	35 (23.5)	9 (33.3)	26 (21.5)	0.21
Ascites	26 (17.4)	8 (29.6)	18 (14.9)	0.092
Gastroesophageal varix	84 (56.4)	14 (51.2)	70 (57.8)	0.52
HGS, kg				
Female	17.4 ± 6.7	14.9 ± 2.2	18.8 ± 8.1	0.32
Male	30.5 ± 9.0	23.6 ± 3.7	31.9 ± 9.1	<0.001
CT-SMI, cm^2^/m^2^				
Female	36.0 ± 6.8	30.4 ± 5.4	39.0 ± 5.6	0.016
Male	46.2 ± 9.6	37.6 ± 4.5	47.8 ± 9.4	<0.001
Hemoglobin, g/dL				
Male	13.1 ± 2.0	11.4 ± 1.6	13.4 ± 1.9	<0.001
Female	11.1 ± 1.9	9.8 ± 1.8	11.8 ± 1.6	0.048
MCV, fL	96.9 ± 9.1	99.5 ± 11.4	96.4 ± 8.6	0.12
MCH, pg	32.0 ± 3.6	32.7 ± 4.3	31.0 ± 3.5	0.28
MCHC, %	33.0 ± 1.1	32.8 ± 1.0	33.0 ± 1.1	0.7
Platelet, ×10⁴/μL	12.7 ± 5.8	13.3 ± 7.6	12.6 ± 5.3	0.55
Albumin, g/dL	3.9 ± 0.6	3.5 ± 0.6	3.9 ± 0.6	<0.001
PT, %	74.9 ± 20.2	69.8 ± 18.1	76.1 ± 20.6	0.15
Total Bilirubin, mg/dL	1.2 (0.9–1.8)	1.2 (0.9–1.8)	1.2 (0.9–1.8)	0.98
ChE, U/L	199.2 ± 84.7	132.0 ± 61.4	214.1 ± 82.4	<0.001
BTR	4.7 ± 1.8	4.4 ± 1.6	4.8 ± 1.8	0.39
NH_3_, μg/dL	49.8 ± 31.3	49.8 ± 23.8	49.8 ± 32.9	0.99
BUN, mg/dL	14.8 ± 6.5	17.9 ± 10.1	14.1 ± 5.1	0.0054
Creatinine, mg/dL	0.82 (0.66–0.99)	0.97 (0.68–1.07)	0.81 (0.66–0.96)	0.074
Sodium, mEq/L	138.8 ± 3.3	137.0 ± 4.2	139.2 ± 3.0	0.0025
Zinc, μg/dL	68.5 ± 16.9	61.7 ± 22.6	70.0 ± 5.2	0.030
EAA	0.30 ± 0.11	0.40 ± 0.12	0.28 ± 0.09	<0.001
P-III-P, U/m	1.0 ± 0.5	1.2 ± 0.5	1.0 ± 0.4	0.0066
Type IV Collagen 7S, ng/mL	8.2 (6.4–11.5)	9.9 (7.1–12.1)	8.0 (6.2–10.6)	0.14
FIB4 index	5.2 ± 3.4	5.8 ± 4.7	5.0 ± 3.1	0.28
Ferritin, ng/mL	105.4 (34.1–241.0)	132.5 (42.0–336.0)	99.0(32.3–209.8)	0.074
CRP, mg/dL	0.12 (0.070–0.60)	0.11 (0.053–0.60)	0.42 (0.10–0.68)	0.48
Myostatin, ng/mL	3.0 ± 1.5	3.3 ± 1.6	2.8 ± 1.4	0.035

Categorical data are presented as number, and continuous data are presented as mean (standard deviation) or median value (interquartile range). ^a^ Comparisons of clinical characteristics between patients with sarcopenia and without sarcopenia were carried out using the χ^2^-test or Student’s *t*-test. BMI, body mass index; HCV, hepatitis C virus; HBV, hepatitis B virus; ALC, alcoholic liver cirrhosis; NASH, nonalcoholic steatohepatitis; HCC, hepatocellular carcinoma; HGS, handgrip strength; SMI, skeletal muscle index; BTR, branched-chain amino acid to tyrosine ratio; BUN, blood urea nitrogen; EAA, endotoxin activity assay; P-III-P, type III procollagen-N-peptide; FIB-4, fibrosis 4 index; MCV, mean corpuscular volume; MCH, mean corpuscular hemoglobin; MCHC, mean corpuscular hemoglobin concentration; PT, prothrombin time; ChE, cholinesterase; NH_3_, ammonia; CRP, C-reactive protein; m-ALBI, modified albumin–bilirubin; CT, computed tomography.

**Table 4:** Clinical characteristics of nonalcoholic patients with sarcopenia and without sarcopenia.

Variables	Nonalcoholic Cirrhosis (Group NA; *n* = 302)	Sarcopenia (Group S; *n* = 71)	Nonsarcopenic (Group N; *n* = 231)	*p*-Value ^a^
Age, years	70.6 ± 9.8	74.4 ± 7.7	69.4 ± 10.1	<0.001
Gender, male/female	153/149	26/45	127/104	0.0096
BMI, kg/m^2^	24.3 ± 4.4	22.1 ± 3.4	25.0 ± 4.5	<0.001
Child–Pugh grade A/B/C	223/66/13	44/19/8	179/47/5	0.0022
Child–Pugh score	6.1 ± 1.5	5.9 ± 1.4	6.6 ± 1.8	0.0011
m-ALBI grade 1/2a/2b/3	133/55/92/22	20/14/27/10	113/41/65/12	0.0042
ALBI score	−2.38 ± 0.63	−2.14 ± 0.64	−2.45 ± 0.61	<0.001
Etiology HCV/HBV/ALC/NASH/others ^b^	126/43/76/57	39/3/11/18	87/40/65/39	<0.001
HCC	70 (23.2)	19 (26.8)	51 (22.1)	0.42
Ascites	53 (15.5)	18 (25.4)	35 (15.2)	0.073
Gastroesophageal varix	140 (46.4)	42 (59.2)	98 (42.4)	0.020
HGS, kg				
Female	16.0 ± 6.3	12.4 ± 3.9	17.5 ± 6.5	<0.001
Male	29.2 ± 8.5	20.7 ± 4.9	31.0 ± 8.0	<0.001
CT-SMI, cm^2^/m^2^				
Female	39.5 ± 8.9	33.7 ± 3.6	42.0 ± 9.3	<0.001
Male	45.9 ± 8.3	37.3 ± 3.4	47.7 ± 7.9	<0.001
Hemoglobin, g/dL				
Male	13.1 ± 2.0	12.0 ± 1.9	13.3 ± 2.0	0.0022
Female	11.9 ± 1.9	11.1 ± 2.2	12.2 ± 1.6	<0.001
MCV, fL	95.5 ± 8.3	95.8 ± 8.0	95.5 ± 8.5	0.81
MCH, pg	31.5 ± 3.2	31.4 ± 3.2	31.5 ± 3.3	0.72
MCHC, %	32.9 ± 0.9	32.7 ± 0.9	33.0 ± 0.9	0.13
Platelet, ×10^4^/μL	11.5 ± 5.4	11.5 ± 5.0	11.5 ± 5.6	0.97
Albumin, g/dL	3.8 ± 0.7	3.5 ± 0.7	3.9 ± 0.6	<0.001
PT, %	77.3 ± 17.6	75.0 ± 19.4	78.0 ± 17.0	0.21
Total bilirubin, mg/dL	1.1 (0.8–1.6)	1.1 (0.8–1.6)	1.1 (0.8–1.6)	0.34
ChE, U/L	205.3 ± 93.9	160.6 ± 73.1	219.0 ± 95.6	<0.001
BTR	4.8 ± 1.9	4.3 ± 1.6	4.9 ± 1.9	0.030
NH_3_, μg/dL	46.1 ± 27.5	46.2 ± 25.6	46.0 ± 28.2	0.96
BUN, mg/dL	17.2 ± 8.2	17.9 ± 8.7	17.0 ± 8.0	0.39
Creatinine, mg/dL	0.78 (0.66–0.97)	0.77 (0.65–0.97)	0.79 (0.66–0.97)	0.95
Sodium, mEq/L	139.1 ± 3.6	138.4 ± 4.6	139.3 ± 3.3	0.075
Zinc, μg/dL	66.8 ± 17.1	62.6 ± 19.3	68.0 ± 16.2	0.024
EAA	0.31 ± 0.14	0.31 ± 0.14	0.30 ± 0.13	0.82
P-III-P, U/m	1.0 ± 0.6	1.2 ± 0.6	1.0 ± 0.6	0.018
Type IV collagen 7S, ng/mL	7.3 (5.6–10.3)	7.1 (5.2–10.3)	7.9 (5.2–11.0)	0.87
FIB4 index	6.1 ± 4.9	6.3 ± 3.5	6.0 ± 5.3	0.65
Ferritin, ng/mL	80.5 (31.8–170.5)	67.1(28.0–156.0)	86.0(31.9–173.9)	0.22
CRP, mg/dL	0.10 (0.020–0.43)	0.18 (0.040–0.78)	0.10 (0.020–0.40)	0.40
Myostatin, ng/mL	3.3 ± 1.1	3.7 ± 1.1	3.0 ± 1.1	0.021

Categorical data are presented as number, and continuous data are presented as mean (standard deviation) or median value (interquartile range). ^a^ Comparisons of clinical characteristics between patients with sarcopenia and without sarcopenia were carried out using the χ^2^-test or Student’s *t*-test. ^b^ Includes autoimmune hepatitis, primary biliary cholangitis. BMI, body mass index; HCV, hepatitis C virus; HBV, hepatitis B virus; ALC, alcoholic liver cirrhosis; NASH, nonalcoholic steatohepatitis; HCC, hepatocellular carcinoma; HGS, handgrip strength; SMI, skeletal muscle index; BTR, branched-chain amino acid to tyrosine ratio; BUN, blood urea nitrogen; EAA, endotoxin activity assay; P-III-P, type III procollagen-N-peptide; FIB-4, fibrosis 4 index; MCV, mean corpuscular volume; MCH, mean corpuscular hemoglobin; MCHC, mean corpuscular hemoglobin concentration; PT, prothrombin time; ChE, cholinesterase; NH_3_, ammonia; CRP, C-reactive protein; m-ALBI, modified albumin–bilirubin; CT, computed tomography.

**Table 5:** Risk factors for sarcopenia in all patients with cirrhosis.

	OR (95% Confidence Interval)	*p* Value	OR (95% Confidence Interval)	*p* Value
Age ≥ 65, Years	3.37 (1.8–6.28)	<0.001	2.39 (1.14–5.01)	0.0021
Sex, male	0.45 (0.29–0.71)	<0.001	0.74 (0.40–1.36)	0.34
Child–Pugh Grade B	1.76 (1.04–2.98)	0.034	0.94 (0.44–1.98)	0.86
Grade C	5.58 (2.22–14.1)	<0.001	3.80 (1.05–13.80)	0.042
ALBI Grade 2	1.75 (0.91–3.37)	0.092		
Grade 2b	2.69 (1.55–4.68)	<0.001		
Grade 3	4.42 (1.95–10.00)	<0.001		
Etiology HBV	2.03 (1.15–3.56)	0.014		
HCV	0.34 (0.098–1.18)	0.085		
NASH	0.77 (0.36–1.64)	0.49		
Other ^a^	1.07 (0.77–1.46)	0.70		
HCC	1.43 (0.87–2.38)	0.16		
Ascites	2.04 (1.19–3.48)	0.0092		
Gastroesophageal Varices	1.41 (0.89–2.22)	0.14		
Hemoglobin < 10.8, g/dL; female		<0.001	4.47 (2.32–8.60)	
Hemoglobin < 12.9, g/dL; male				
Platelet < 12, 10⁴/μL	1.32 (0.83–2.09)	0.24		
Albumin < 3.5, g/dL	2.54 (1.59–4.06)	<0.001		
Prothrombin Time < 70, %	1.42 (0.90–2.25)	0.13		
Total Bilirubin > 1.5, mg/dL	0.93 (0.57–1.53)	0.78		
Cholinesterase < 200, U/L	3.30 (2.02–5.39)	<0.001	1.87 (0.87–4.05)	0.11
BTR < 5	1.61 (0.99–2.60)	0.054		
Ammonia > 50, μg/dL	1.25 (0.77–2.03)	0.36		
BUN > 16, mg/dL	1.44 (0.92–2.27)	0.11		
Creatinine > 0.8, mg/dL	1.00 (0.64–1.56)	0.99		
Sodium < 138, mEq/L	1.52 (0.93–2.49)	0.095		
Zinc < 60, μg/dL	1.69 (1.04–2.74)	0.034	1.07 (0.51–2.25)	0.85
EAA > 0.4	2.33 (1.33–4.08)	0.0032	1.67 (0.91–3.07)	0.10
P-III-P > 1, U/mL	1.90 (1.16–3.11)	0.011	0.98 (0.50–1.91)	0.95
7S domain of type IV collagen > 8, ng/mL	1.41 (0.87–2.30)	0.16		
FIB4 index > 5	1.52 (0.97–2.39)	0.067		
Ferritin ≥ 200, ng/mL	1.36 (0.81–2.26)	0.24		
CRP > 0.10, mg/dL	1.55 (0.97–2.46)	0.065		
Myostatin > 3.1, ng/mL	1.60 (0.95–2.02)	0.071		

^a^ Includes autoimmune hepatitis, primary biliary cholangitis. OR, odds ratio; BMI, body mass index; HBV, hepatitis B virus; HCV, hepatitis C virus; NAFLD, nonalcoholic fatty liver disease; HCC, hepatocellular carcinoma; HGS, handgrip strength; SMI, skeletal muscle index; BTR, branched-chain amino acid to tyrosine ratio; BUN, blood urea nitrogen; EAA, Endotoxin Activity Assay; P-III-P, type III procollagen-N-peptide, FIB4 index, fibrosis-4 index.

**Table 6:** Risk factors for sarcopenia in patients with alcoholic cirrhosis.

	Univariate Analysis		Multivariate Analysis	
	OR (95% Confidence Interval)	*p* Value	OR (95% Confidence Interval)	*p* Value
Age ≥ 65, Years	2.05 (0.81–5.22)	0.13		
Sex, male	0.35 (0.11–1.15)	0.083		
Child–Pugh Grade B	2.07 (0.79–5.41)	0.14		
Grade C	4.45 (0.91–21.8)	0.065		
ALBI Grade 2	1.58 (0.44–5.62)	0.48		
Grade 2b	3.84 (1.33–11.1)	0.013		
Grade 3	3.93 (0.80–19.30)	0.092		
HCC	1.85 (0.743–4.59)	0.19		
Ascites	2.41 (0.92–6.33)	0.074		
Gastroesophageal Varices	0.74 (0.32–1.71)	0.48		
Hemoglobin < 10.8, g/dL; female	8.76 (3.37–22.80)	<0.001	5.97 (1.88–19.00)	0.0025
Hemoglobin < 12.9, g/dL; male				
Platelet < 12, 10⁴/μL	1.29 (0.56–2.99)	0.55		
Albumin < 3.5, g/dL	3.6 (1.50–8.63)	0.0043		
Prothrombin Time < 70, %	1.75 (0.75–4.06)	0.20		
Total Bilirubin > 1.5, mg/dL	1.25 (0.53–2.98)	0.61		
Cholinesterase < 200, U/L	6.56 (2.14–20.10)	<0.001	2.13 (0.57–7.93)	0.26
BTR < 5	1.39 (0.577–3.36)	0.46		
Ammonia > 50, μg/dL	1.52 (0.64–3.64)	0.34		
BUN > 16, mg/dL	1.78 (0.77–4.12)	0.18		
Creatinine > 0.8, mg/dL	1.41 (0.60–3.28)	0.43		
Sodium < 138, mEq/L	2.80 (1.17–6.70)	0.021		
Zinc < 60, μg/dL	3.75 (1.51–9.32)	0.0044		
EAA > 0.4	10.4 (3.62–29.90)	<0.001	5.02 (1.60–15.80)	0.0057
P-III-P > 1, U/mL	2.10 (0.85–5.19)	0.11		
7S domain of type IV collagen > 8, ng/mL	1.91 (0.75–4.87)	0.18		
FIB4 index > 5	1.15 (0.48–2.73)	0.76		
Ferritin ≥ 200, ng/mL	1.75 (0.88–3.51)	0.11		
CRP > 0.10, mg/dL	2.09 (0.83–5.23)	0.10		
Myostatin > 3.1, ng/mL	1.57 (0.68–3.09)	0.22		

OR, odds ratio; BMI, body mass index; HCC, hepatocellular carcinoma; HGS, handgrip strength; SMI, skeletal muscle index; BTR, branched-chain amino acid to tyrosine ratio; BUN, blood urea nitrogen; EAA, endotoxin activity assay; P-III-P, type III procollagen-N-peptide; FIB4 index, fibrosis-4 index.

**Table 7:** Risk factors for sarcopenia in patients with nonalcoholic cirrhosis.

	OR (95% Confidence Interval)	*p* Value	OR (95% Confidence Interval)	*p* Value
Age ≥ 65, Years	4.61 (1.91–11.20)	<0.001	3.09 (1.21–7.87)	0.018
Sex, male	0.47 (0.27–0.82)	0.0074	0.91 (0.46–1.77)	0.77
Child–Pugh Grade B	1.64 (0.88–3.08)	0.12	0.77 (0.33–1.80)	0.55
Grade C	6.51 (2.03–20.9)	0.0016	4.10 (1.09–15.50)	0.037
ALBI Grade 2a	1.93 (0.89–4.17)	0.095		
Grade 2b	2.35 (1.22–4.51)	0.011		
Grade 3	4.71 (1.79–12.4)	0.0016		
Etiology HCV	5.98 (1.74–20.5)	0.0045		
NASH	2.26 (0.59–8.58)	0.23		
other ^a^	6.15 (1.68–22.6)	0.0061		
HCC	1.29 (0.70–2.37)	0.41		
Ascites	1.90 (0.99–3.62)	0.051		
Gastroesophageal Varices	1.91 (1.11–3.30)	0.020	2.11 (1.06–4.20)	0.033
Hemoglobin < 10.8, g/dL; female	5.15 (2.88–9.21)	<0.001	5.34 (2.52–11.30)	<0.001
Hemoglobin < 12.9, g/dL; male				
Platelet < 12, 10^4^/μL	1.29 (0.74–2.23)	0.37		
Albumin < 3.5, g/dL	2.18 (1.25–3.81)	<0.001		
Prothrombin Time < 70, %	1.33 (0.76–2.31)	0.32		
Total Bilirubin > 1.5, mg/dL	0.83 (0.45–1.52)	0.55		
Cholinesterase < 200, U/L	2.75 (1.57–4.83)	<0.001	1.34 (0.60–2.99)	0.48
BTR < 5	1.73 (0.97–3.08)	0.062		
Ammonia > 50, μg/dL	1.18 (0.66–2.12)	0.57		
BUN > 16, mg/dL	1.32 (0.77–2.25)	0.31		
Creatinine > 0.8, mg/dL	0.89 (0.52–1.52)	0.99		
Sodium < 138, mEq/L	1.25 (0.67–2.33)	0.47		
Zinc < 60, μg/dL	1.44 (0.81–2.55)	0.22		
EAA > 0.4	1.19 (0.59–2.42)	0.63		
P-III-P > 1, U/mL	1.83 (1.01–3.31)	0.045	1.09 (0.52–2.29)	0.82
7S domain of type IV collagen > 8, ng/mL	1.32 (0.74–2.36)	0.34		
FIB4 index > 5	1.63 (0.95–2.80)	0.076		
Ferritin ≥ 200, ng/mL	0.90 (0.46–1.76)	0.75		
CRP > 0.10, mg/dL	1.45 (0.83–2.53)	0.19		
Myostatin > 3.1, ng/mL	1.65 (0.92–2.76)	0.084		

^a^ Includes autoimmune hepatitis, primary biliary cholangitis. OR, odds ratio; BMI, body mass index; HBV, hepatitis B virus; HCV, hepatitis C virus; HCC, hepatocellular carcinoma; HGS, handgrip strength; SMI, skeletal muscle index; BTR, branched-chain amino acid to tyrosine ratio; BUN, blood urea nitrogen; EAA, endotoxin activity assay; P-III-P, type III procollagen-N-peptide; FIB4 index, fibrosis-4 index.

## Data Availability

Raw data were generated at Nara Medical University Hospital. Derived data supporting the findings of this study are available from the corresponding author (T.N.) on request.
